# Tetanus

**Published:** 1913-05

**Authors:** G. B. Taylor

**Affiliations:** Cameron, Texas


For Texas Medical Journal.

### Tetanus.

BY G. B. TAYLOR, M. D., CAMERON, TEXAS.

*History.*—The patient, a white boy, aged 16, was brought to my home, December 13, about 8 p. m., complaining of a slight pain between his shoulder blades, some sore throat, which gave him some pain to swallow, and could open his mouth about half. Some ten days before coming to see me, while out in the lot attending to his stock, he stepped on an old rake that they used in cleaning up the premises and lot. One of the points of the rake stuck through his rubber boot and sock, going into the arch of his left foot, about one-half inch. He came back to the house and told his parents what had happened to him. His mother having some bichloride tablets, she made up a solution of about one to six thousand, and began to bathe the foot, two and three times daily, for three or four days. His foot healed up externally and they thought he was well, but as he was attending school, for the first two or three days it was necessary for him to use a crutch. After that time he began to wear his shoe with comfort.

*Physical Examination.*—December 13, 8 p. m., I found him to have a very foul tongue, temperature normal, respiration normal, pulse 85, patella reflex very acute, pupils responded very quick, kidneys acting good but constipated, some pain on pressure of the muscles of the neck, on either side. Had him remove his shoe to examine his foot, it had healed externally, no redness nor pain on pressure. I had him taken home and put to bed, and started up a brisk purgative of calomel.

Next morning, December 14, I told his parents he had a case of tetanus, and that I was going to administer the tetanus serum, and wanted some other doctors to help me with the case. I had two other doctors called in and they agreed with me, and we started up our serum. First serum given December 14, about 9 p. m., 6,000 c.c., all we had our hands on, subcutaneously, and opened up the wound on foot and found amount of pus. One hour after giving the serum his temperature ran up about two degrees, remained on him about two hours and abated down to normal. His pulse at this time was 120, and respiration 20, not complaining of much pain anywhere; jaw about the same condition as night before, but abdominal muscles were beginning to get rigid, bowels and kidneys acting good.

December 14, at about 7 p. m., I gave him 6,000 c.c., subcutaneously and his temperature ran up about 2 degrees, one hour after giving the serum, and lasted about one hour and abated down to normal. As he was getting a little more nervous, I gave him 2 drams of bromidia in about 2 ounces of sweet milk, per rectum; pulse standing all the time at 120 and respiration about 22.

December 14, at about 10 p. m., I gave him 10,000 c.c., in the veins, about one hour after the administration of the serum, his temperature ran up about 3 degrees, while his temperature would go up, it did not seem to have any affect on his circulation or respiration, as both standing at same for last 8 or 12 hours, but inclined to get a little nervous all the time, though conscious all the time and claimed more of getting tired of the bed than anything else.

December 15, at about 9 a. m., I gave him 10,000 c.c., in the veins, it having the same affect on his temperature as previus doses, but not affecting his circulation or his breathing; just a little more nervous and some twitching of the facial muscles at times. I gave him one-quarter of a grain of morphine hypodermically, he drinking plenty of water and sweating freely; the kidneys acting good, and taking orange juice with egg albumin every two or three hours.

December 15, at about 6 p. m., I gave him 20,000 c.c., in the veins, and started up the normal saline solution by rectum, he was more restless than any time before and I gave him about 30 grains of bromide and about 10 grains of chloral by the stomach. First medicine administered by the stomach, and that seemed to have a nice effect for about one hour. Pulse standing at about 125 and his respiration running about 25.

December 16, at about 1 a. m., I gave him 10,000 c.c., in the veins, his pulse standing at this time about 130 and respiration about 30 and spasms began to be more noticeable. At about this time I gave him one-quarter of a grain of morphine and 1-100 grain hyoscyamine. This dose of serum causing same rise of temperature as previous.

December 16, at about 9 a. m., I gave him 10,000 c.c., in the veins again, his pulse at this time being about 130 and his respiration about 35, and getting very laborious, and was necessary to give him another dose of morphine hypodermically, paroxysms getting some stronger all the time.

December 16, at about 3 p. m., I gave him the last dose, 10,000 c.c., in the spine, taking off about two ounces of the spinal fluid, which did not look bad, but from the flow, seemed to be great pressure. At the time of administering this dose in the spine, his temperature was 103, pulse 165, respiration 50 in a few minutes following this dose of serum, he had one convulsion that drew him about half back, and from that time on I kept him under chloroform. He died at 6 p. m., and by failure of respiration, as I could feel his heart beat a few seconds after respiration quit. I took his temperature about 10 minutes before death, under his arm and it registered 108. Total amount of serum used 82,000 c.c., and being my first tetanus serum to use, I want to know if it causes an elevation of temperature in every case as it did in mine.

